# Some Compositional and Kinetic Controls on the Bioenergetic Landscapes in Oceanic Basement

**DOI:** 10.3389/fmicb.2016.00107

**Published:** 2016-02-09

**Authors:** Wolfgang Bach

**Affiliations:** ^1^MARUM and Geoscience Department, University of BremenBremen, Germany; ^2^Centre of Excellence in Geobiology and Department of Earth Sciences, University of BergenBergen, Norway

**Keywords:** ocean crust, ridge flanks, seawater circulation, water–rock interactions, subseafloor life, bioenergetics

## Abstract

This contribution assesses the availability of catabolic energy for microbial life during water–rock reactions in the flanks of mid-ocean ridges, where basaltic and ultramafic rocks interact with circulating seawater. In addition to equilibrium thermodynamic computations, results for kinetic reaction paths are presented. In these calculations, it is assumed that dissolution of olivine and basalt glass control the rates of hydrogen forming reactions in ultramafic and basaltic rocks, respectively. The results suggest that all ocean crust basement rocks release enough hydrogen (H_2_,aq) to support hydrogenotrophic life at low water-to-rock ratios. Olivine dissolution rate control imposes a stronger effect on hydrogen production than phase equilibrium controls, indicating that magnetite formation is not a requirement for production of large amounts of hydrogen in ultramafic rocks. The formation of non-tronite and celadonite are primarily responsible for the formation of the moderate amounts of hydrogen (H_2_,aq) expected in basaltic ridge flanks. Under conditions of large seawater fluxes required to account for the great global convective heat flow in ridge flanks, however, hydrogen production in basaltic ridge flanks is insufficient for supporting hydrogenotrophic life. It is hence proposed that the role of Fe oxidation in basaltic ridge flanks is greater than previously suggested. A standing stock of 2.4^∗^10^28^ cells may be supported by Fe oxidation in basaltic ridge flanks, equivalent of about 10% of the sedimentary deep biosphere. The size of a hydrogenotrophic biomass within the ocean crust is more difficult to estimate because the rates and processes of hydrogen release are insufficiently constrained. In any case, hydrogenotrophy in the ocean crust should be of key importance only in olivine-rich basement rocks and in sedimented ridge flanks with low time-integrated seawater fluxes.

## Introduction

Seawater flows in aquifers within the seafloor at rates so large that it takes only few 100s of 1000s years to process the entire volume of the oceans through the permeable ocean crust (e.g., Elderfield and Schultz, 1996; Fisher, 2005). This tremendous flux, coupled with exchange reactions between the crust and the circulating seawater, is critical in global budgets of ocean-lithosphere exchange. Important types of reactions include the removal of Mg^++^ and HCO_3_^−^ by minerals (smectite, carbonate) that fill fracture and void space within the crust. Alteration of basaltic glass removes Ca^++^ and SiO_2_ from the crust into the oceans, where these components are taken up in algal and protozoan tests. These processes play a central role in the silicate–carbonate loop of the Earth’s carbon cycle (e.g., Arvidson et al., 2006). Alteration reactions also consume oxygen, which makes them a crucial sink for oxidizing power that is continuously produced in the carbon cycle by burial of organic matter (Hayes and Waldbauer, 2006). In addition, the ocean crust may harbor microbial life, which possibly impacts these global scale carbon and redox budgets. Perhaps more importantly, microbial activity within the ocean crust likely affects the rates and pathways of reactions governing exchange between seawater and oceanic basement.

Most of the seawater circulation takes place in ridge flanks, where temperatures are low and the rates of water–rock reaction are slow. The low to moderate temperatures in the ridge flank settings and the sluggish kinetics of abiotic water–rock reactions allow chemolithoautotrophic microorganisms to harness the Gibbs energy (Δ_r_G) associated with redox reactions during alteration. An initial appraisal of the potential size of biomass living in these rocky habitats suggests that the cell numbers may resemble those in the sedimentary deep biosphere (Bach and Edwards, 2003).

The principal energy sources (electron donors) are divalent iron (Fe^+2^) and sulfide (S^−2^) dissolved in basaltic glass or forming minerals in volcanic and mantle rocks. These reduced constituents of rocks are oxidized in alteration reactions. If the interacting fluid is oxygenated seawater, oxygen is consumed. Different electron acceptors (NO_3_^−^, SO_4_^2−^, HCO_3_^−^, ferric hydroxide, etc.) are used in anoxic ridge flank environments (Boettger et al., 2013). Tectonic denudation of olivine-dominated rocks (dunites, peridotites, troctolites) are common along mid-ocean ridges and as much as 50% of the seafloor created along slow and ultraslow spreading ridges may expose these rock types (Escartin et al., 2008; Cannat et al., 2010). Olivine-rich rocks undergo fairly rapid reactions with seawater (serpentinization), during which hydrogen (H_2_,aq) may form (McCollom and Bach, 2009; Nakamura et al., 2009; Klein et al., 2013). The reducing potential of micromolal quantities of H_2_ is large enough to drive *e*^−^ transfers to all electron acceptors, including CO_2_. Again, these transfers are sluggish in the abiotic world at low temperatures, but H_2_ is turned over very quickly by microorganisms under these conditions (e.g., Hoehler et al., 2001). High hydrogen-yields in serpentinization are contrasted by very low H_2_ production related to radiolysis in the subseafloor. But even such small quantities of H_2_ are utilized by aerobic microorganisms in energy-starved sedimentary environments with exceedingly low flux of reducing power (Blair et al., 2007).

In this communication, I examine some energetic constraints pertinent to the potential abundance and functioning of microbial life in the oceanic basement (Orcutt et al., 2011; Edwards et al., 2012), where basalt and peridotite are the principal sources of reducing power. In the kinetics calculations, I focus on reactions involving basaltic glass and olivine, as rate data are available for these two substrates, which are abundant phases in volcanic and ultramafic ridge flank environments, respectively.

## Materials and Methods

**FIGURE 1:**
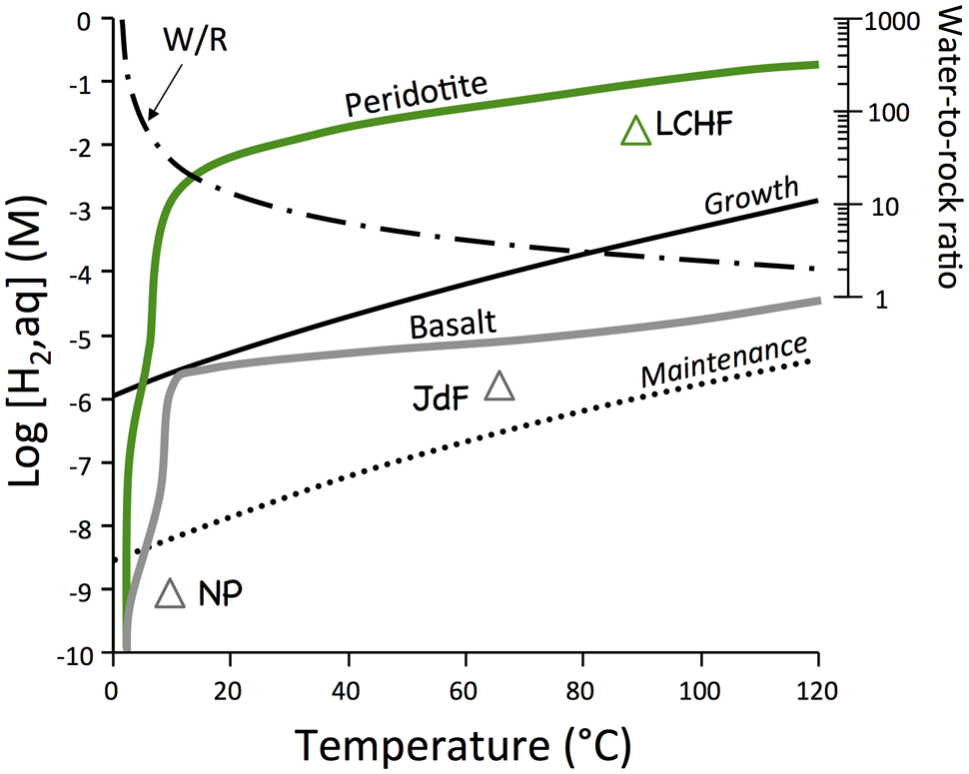
**Equilibrium thermodynamic calculation of potential hydrogen supply by water–rock reactions and hydrogen demand by hydrogenotrophic microorganisms (Hoehler, 2004)**. Off-axis systems in basalt (NP = North Pond; JDF = Juan de Fuca ridge flank) in gray and the Lost City Hydrothermal Vent Field (LHVF) are also shown. Calculations were conducted with EQ3/6 along a path that had water–rock ratios decrease from infinity to two as temperatures increased from 2 to 120°C.

Thermodynamic reaction path modeling using both arbitrary and true kinetics mode was conducted to examine the roles of substrate composition, secondary mineral compositions, and rates of primary mineral dissolution on the extent and timing of hydrogen (H_2_,aq) release from water rock reactions in the seafloor. The thermodynamic calculations of hydrogen yields were conducted using the EQ3/6 code (Wolery and Jarek, 2003) if solid solution compositions were permitted. Ideal solid solution compositions as in Klein et al. (2013) were allowed to form in these reaction paths, in which rock was added incrementally to decrease water–rock ratios. The REACT code of Geochemist’s Workbench (GWB; Bethke, 1996) was used for computing most of the kinetic reaction path models in which basalt glass or olivine dissolution was the rate-limiting step. I used 500-bar databases compiled from SUPCRT92 (Johnson et al., 1991) for these model calculations. Included in the GWB database was mid-ocean ridge basalt glass as a phase, for which thermodynamic properties were calculated with entropy, volume, and free energy data from Chermak and Rimstidt (1989) and Holland (1989) using a polyhedral approach (cf. Oelkers and Gislason, 2001). The composition of the glass (Ca_0.25174_Na_0.0977_K_0.0013_Fe^++^_0.1464_Fe^+++^_0.01627_Mg_0.2585_ Al_0.3844_Si_1.0000_ O_3.3566_) was calculated from average oxide concentrations of mid-Atlantic Ridge basalt reported by Klein (2004). The same basalt composition – added as “special reactant” – was used in the EQ6 model run presented in **Figure 1**. Also included were secondary Fe-phases, such as Fe–Al celadonite, ripidolite, and goethite (Wolery, 2004). Basalt glass dissolution rates at 25°C of 10^−15^ mol cm^−2^ s^−1^ (Oelkers, 2001) and 10^−7^ mol cm^−2^ s^−1^ at 90°C (Daux et al., 1997) were used to estimate a 10°C rate of 10^−16^ mol cm^−2^ s^−1^. Olivine dissolution rates of 10^−14^ mol cm^−2^ s^−1^ for 25°C and neutral pH were taken from Pokrovsky and Schott (2000). A pH-dependency of reaction rates was not applied. Arrhenius parameters for dissolution of olivine, orthopyroxene, and clinopyroxene were provided in Seyfried et al. (2007). The peridotite composition used in the EQ6 thermodynamic calculations was assumed 70% olivine, 25% orthopyroxene, and 5% clinopyroxene, with X_Mg_ set to 0.9 in all these phases. A specific surface area (SSA) of 1 m^2^ g^−1^ was assumed in all kinetics calculations, as it may serve as good approximation for the SSA of fresh basaltic crust (Nielsen and Fisk, 2010).

It should be noted that the presented results of predicted hydrogen levels have large uncertainties due to poorly known thermodynamic properties for some secondary minerals and largely unknown concentration-activity relations in the solid solution phases at low temperatures. The effect of variations in pressure and primary mineral compositions is small by comparison. The calculation results can hence be applied to seafloor systems with <5000 m water depth (i.e., 500 bar pressure) and to basalt and peridotite with compositions that deviate slightly from those used in the calculations. The time values in the true kinetics calculations are plagued by an even larger ambiguity, because the uncertainties in the rate constants and the SSAs of fractured rocks are very large. For instance, if average SSAs of partially altered oceanic crust of 3.4 m^2^ g^−1^ (Nielsen and Fisk, 2010) were used, all calculated rates would be 3.4 times faster. Therefore, the true kinetics calculation results merely have order-of-magnitude-type accuracies as far as predicted time scales are concerned.

## Results

### Equilibrium Thermodynamic Computations of Hydrogen Release

The first set of computations examines the differences in the hydrogen generating potentials between basalt and peridotite, the two most abundant rock types in the oceanic basement. The reaction paths were selected such that recharge of seawater into basement and concomitant heating of the water is represented. Consequently, temperatures go up along the reaction paths as water-to-rock ratios decrease. The results of these computations are compared with the demands of hydrogen in maintenance and growth of hydrogenotropic microbial communities (Hoehler, 2004). The results presented in **Figure 1** indicate that basalt–seawater reactions in off-axis environments can provide enough hydrogen for maintenance of hydrogenotrophic communities. Peridotites are predicted to produce hydrogen levels that are 3 to 4 orders of magnitude greater than during basalt–seawater interactions under identical conditions of temperature and water-to-rock ratios. These quantities of hydrogen are sufficient to allow growth of hydrogenotrophic communities across a large range of temperatures.

### Kinetic–Thermodynamic Computations of Hydrogen Release

A number of computations were conducted to examine the temporal evolution of water–rock systems assuming that the dissolution of mafic minerals in a peridotite and glass in the basaltic crust are the rate-limiting step in the overall reactions.

**FIGURE 2:**
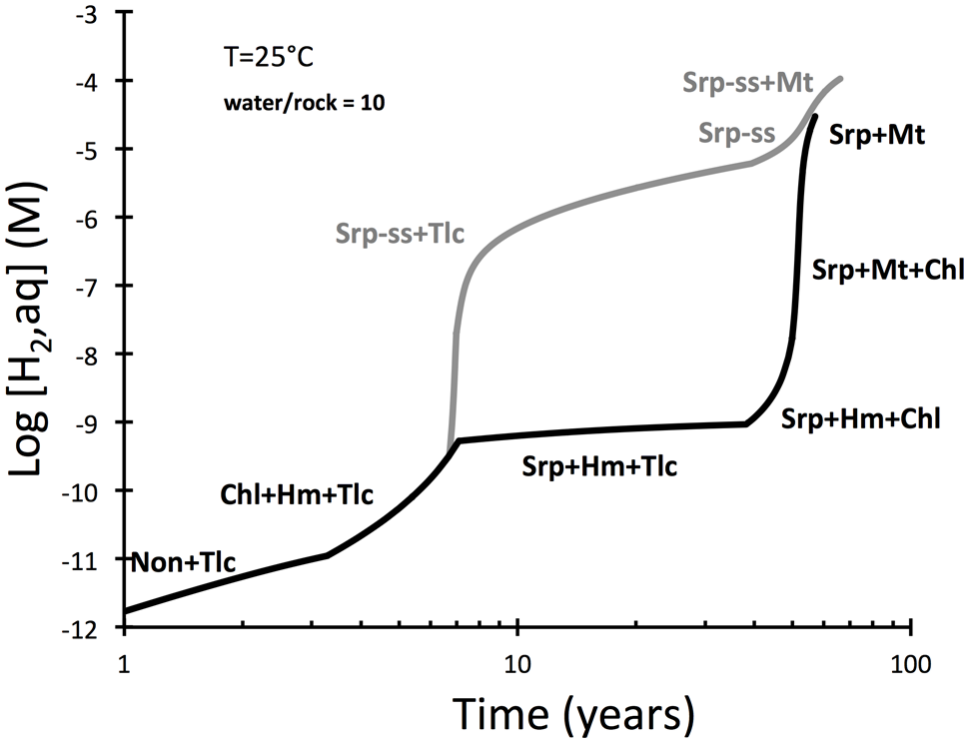
**Predicted hydrogen yields during serpentinization of peridotite and considering dissolution rates of olivine and pyroxenes (EQ6)**. The upper curve represents results of a calculation that allowed serpentine solid solutions (serp-ss) to form. Those were not allowed to from in a second calculation represented by the lower curve. Talc formation early in the reaction path is from loss of silica dissolved in seawater to the rock. Note that the curves merge when magnetite is predicted to become stable.

Production of hydrogen during low-temperature interactions between seawater and peridotite were examined in **Figure 2**. The evolution of hydrogen in the intergranular fluid of peridotite is predicted to increase in a step-wise fashion, from concentrations <1 nM in the first years to concentration >10 μM after more than 50 years. An interesting discrepancy develops in the period 5 to 50 years between a model run in which serpentine solid solutions were allowed and another run that had solid solutions suppressed. These results suggest that the formation of serpentine with ferric iron dissolved in the tetrahedral and octahedral sites may facilitate rapid generation of hydrogen.

**FIGURE 3:**
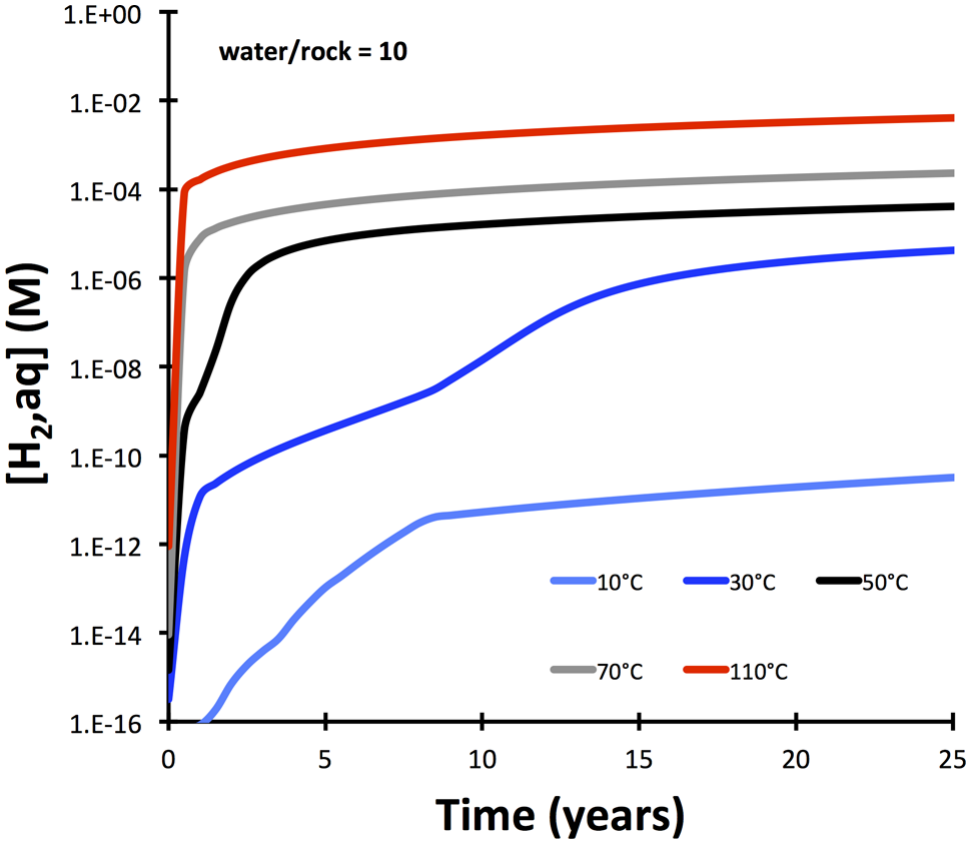
**Temperature dependency of hydrogen release from serpentinization of olivine (Fo90) with dissolution of olivine as rate-limiting step**. The model (calculated with GWB) is simplified compared to the one presented in **Figure 2** in that solid solutions are not allowed.

Hydrogen production is also strongly dependent on temperature and is predicted to change from sluggish at 10°C to rapid at 110°C (**Figure 3**). These predicted fast rates at temperatures of 50°C and greater are inconsistent with experimental results (Mayhew et al., 2013), indicating that either dissolution rates of olivine are over-predicted at higher temperatures by the model or that processes other than olivine dissolution are rate-limiting.

**FIGURE 4:**
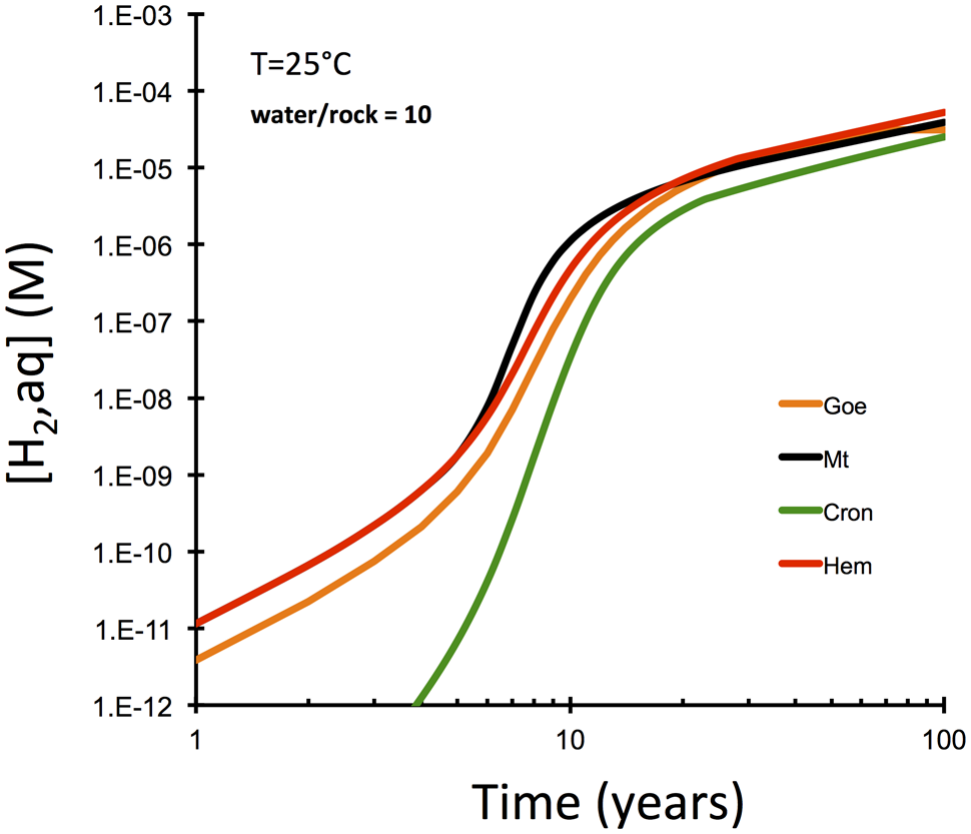
**Computational results for olivine-dissolution controlled serpentinization kinetics examining the role of the nature of secondary Fe(III) phase (GWB)**. Note that magnetite does not produce hydrogen faster than other oxide/oxyhydroxides along this kinetically controlled reaction path.

Another set of reaction paths were computed to investigate the role of the nature of secondary Fe(III) mineral phase in hydrogen production (**Figure 4**). The interesting observation here is that the type of secondary Fe(III) mineral is only of secondary importance, if olivine dissolution is the rate-limiting step. These results suggest that magnetite is not required to explain hydrogen production. More oxidized phases like hematite and goethite can cause just as much hydrogen production in the kinetically controlled intial phase of peridotite–water interaction. Hydrogen production is predicted to be somewhat retarded, when oxides are not allowed to form and Fe(III)-bearing serpentine (cronstedtite, Mg_2_Fe^II^SiFe^III^O_5_(OH)_4_) forms instead.

**FIGURE 5:**
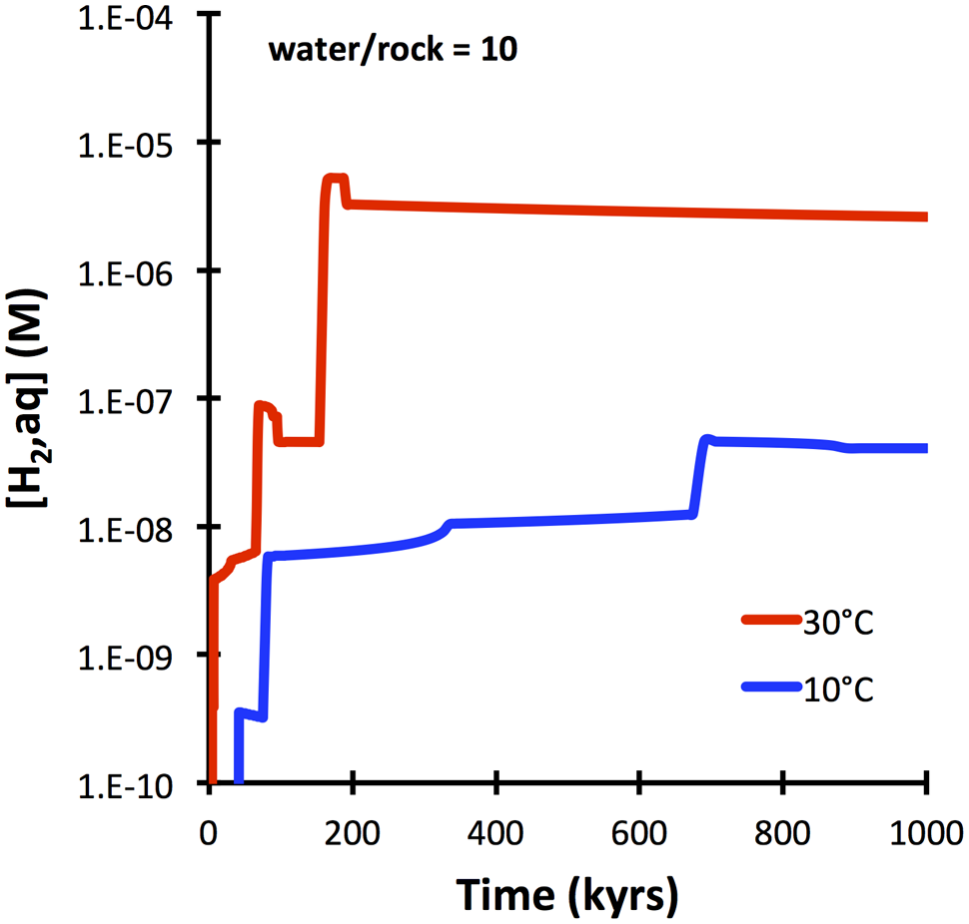
**Hydrogen production predicted for basalt-seawater interactions at 10 and 30°C (GWB)**. Kinetics is controlled by dissolution of basalt glass with an SSA of 1 m^2^ g^−1^ (see text).

Basalt glass is predicted to dissolve more slowly (10^−15^ mol cm^−2^ sec^−1^) than olivine (10^−14^ mol cm^−2^ sec^−1^ at 25°C). It is also expected to yield overall lower amounts of aqueous hydrogen upon water–rock reactions when compared with olivine-rich lithologies (**Figure 1**). The predicted evolution of hydrogen in waters interacting with basalt glass at 10 and 30°C is shown in **Figure 5**. Although slower than peridotite (cf. **Figure 3**), basalt glass is predicted to produce nanomolal to micromolal quantities of aqueous hydrogen in the course of a 100,000 years.

**FIGURE 6:**
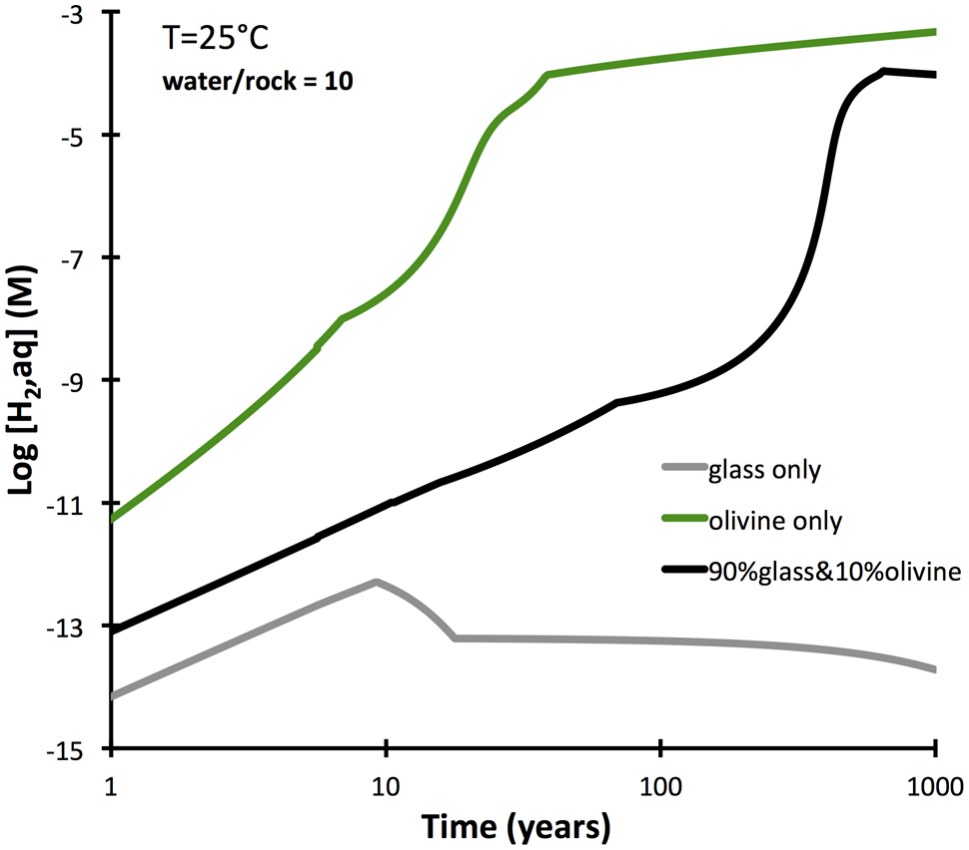
**Dissolution-controlled kinetic reaction paths from pure olivine (dunite) and basalt glass in comparison with an olivine basalt, composed for 90% basalt glass and 10% olivine (Fo_80_)**. Calculations were conducted by GWB, and forsterite dissolution rates are used.

Basaltic rocks often have olivine crystals distributed within a glassy mesostasis. An additional set of reactions paths were computed to determine the difference between olivine-bearing glassy basalt and pure basalt glass in the intial 1000 years of alteration. The results (**Figure 6**) show that a moderate proportion of olivine (10%) in a glassy basalt does increase the predicted hydrogen production markedly relative to olivine-free basalt, although it falls short of that predicted for an olivine rock (dunite). The increased hydrogen production is due to the faster dissolution rate of olivine compared to the glass. If the two phases dissolved at the same rate, the presence of olivine in the basalt would not increase hydrogen production at all, since ferroan smectite (and not serpentine and oxide) is predicted to form.

**FIGURE 7:**
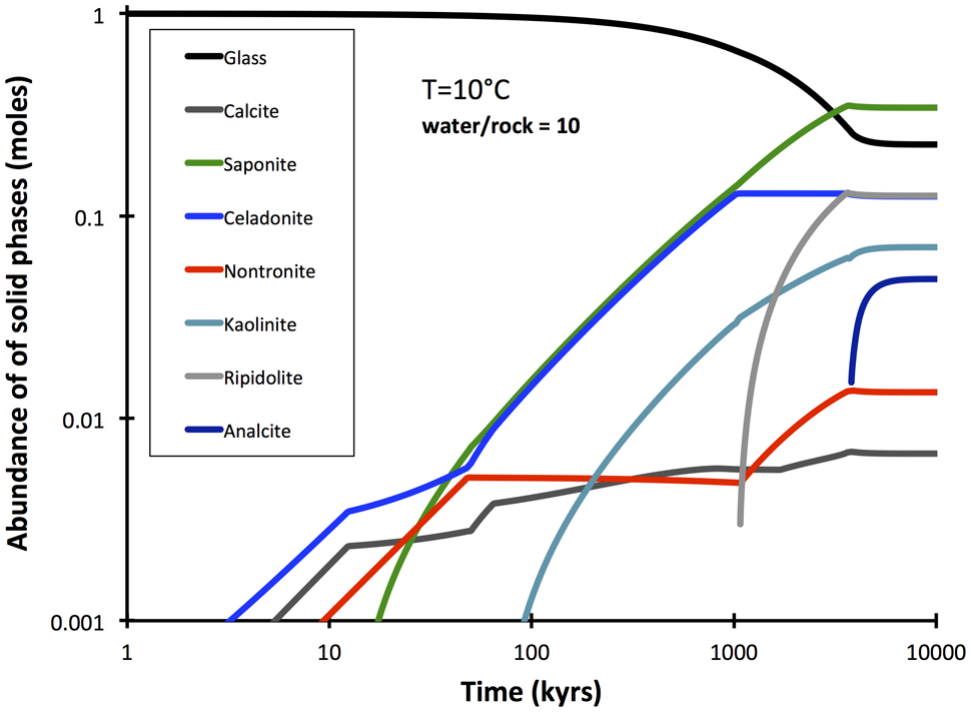
**Predicted reaction extent and products in the course of 10 Myrs of alteration at low water-to-rock ratios (10)**. The computation was conducted with GWB.

**FIGURE 8:**
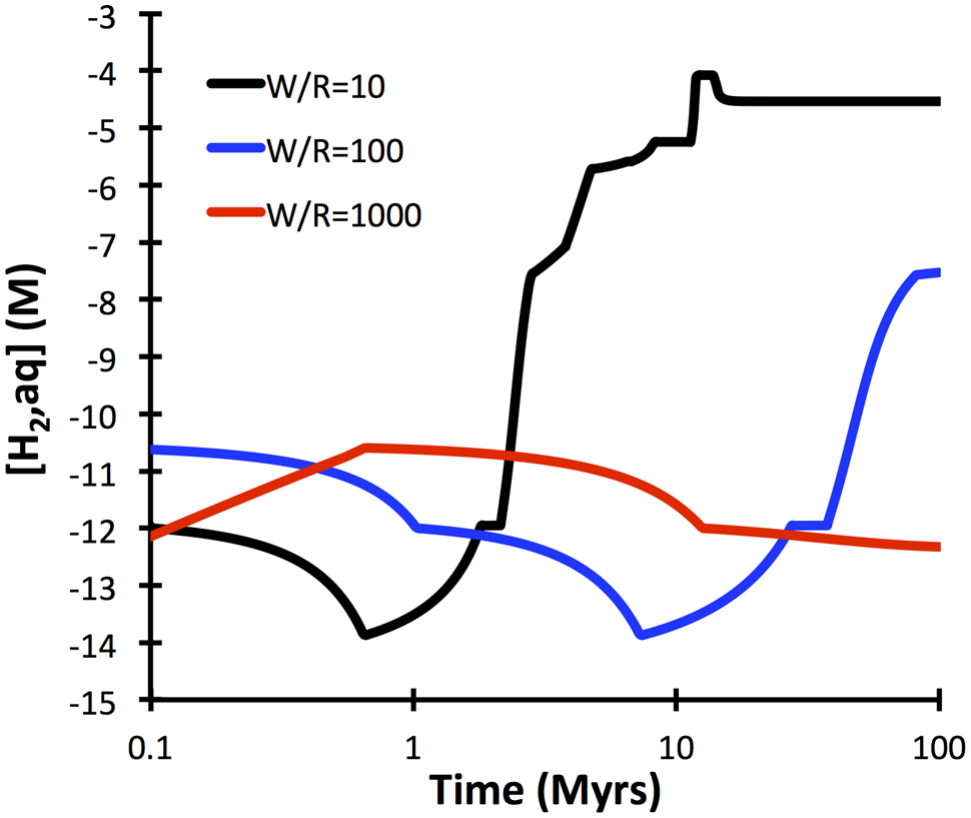
**Dependency of hydrogen release kinetics as a function of water-to-rock ratios**. Note that at high water-to-rock ratios hydrogen concentrations >1 nM are not predicted to develop. The computations were conducted with GWB for a temperature of 10°C.

A comprehensive reaction path model was computed for low-temperature (10°C) alteration of basalt glass over the course of 10 million years (**Figure 7**). The model predicts that non-tronite (Na_0.33_Fe^III^_2_Al_0.33_Si_3.67_O_10_(OH)_2_)^∗^nH_2_O and celadonite (KAlFe^III^Si_4_O_10_(OH)_2_) drive most of the hydrogen production. Predictions of the quantities of hydrogen in ridge flanks depend heavily on the assumed fluxes (time-integrated water-to-rock ratios). High water-to-rock ratios (e.g., open circulation) does not result in noticable accumulations of hydrogen in volcanic ridge flank settings (**Figure 8**).

## Discussion

I examined water–rock reactions and energetic implications for related microbial life in three different ridge flank (*sensu lato*) habitats: (i) open circulation of oxygenated seawater (e.g., the North Pond site; Edwards et al., 2012; Orcutt et al., 2013), (ii) closed-system circulation of suboxic to anoxic seawater (eastern flank of the Juan de Fuca Ridge; Fisher et al., 2005), and (iii) seawater interaction with mantle peridotite in fracture zones and off-axis oceanic core complexes (e.g., Kelley et al., 2001). The effects of variable rock composition (e.g., basaltic glass vs. olivine-phyric basalt vs. peridotite) are examined as well as the consequences of variable dissolution and precipitation reactions.

Dissolved hydrogen (H_2_,aq) has long been known to be one of the most potent energy sources for chemolithoautotrophic microorganisms in the deep sea (e.g., Edwards et al., 2005). It has been demonstrated that different rock types have strongly variable abilities to release hydrogen upon water–rock reactions. It has been shown that peridotites can generate 100s of millimoles of hydrogen per kg of water in the course of high-temperature (200–300°C) serpentinization reactions (e.g., McCollom and Bach, 2009). The results presented above show that peridotites have much greater potential for producing hydrogen than basalts. This difference may account for the fact that isotopic evidence for sulfate reduction is very commonly found in mantle peridotites (e.g., Alt and Shanks, 1998, 2003; Alt et al., 2007) than in basalt. The hydrogen contents of intergranular fluids during serpentinization are high enough under a large range of conditions, even at low temperatures, to allow for hydrogenotrophic sulfate reductions. The drive for hydrogenotrophic sulfate reduction is smaller in basalt systems, but may facilitate maintenance of sulfate reducing communities. The sulfate-reducing bacteria identified in the Juan de Fuca Ridge flank (Lever et al., 2013) may hence be supported by hydrogen (Boettger et al., 2013), although it is also possible that organic carbon compounds are used as electron donor.

Equilibrium thermodynamic predictions of hydrogen yields during low-temperature water–rock reactions are of limited use, because these reactions commonly do not reach the state of equilibrium due to sluggish reaction kinetics. We do not currently have a good understanding of what the rate-limiting steps in production of hydrogen in water–rock reactions are. Most of the calculations presented above work on the assumption that olivine dissolution is an important rate-limiting in the overall reactions that release hydrogen. However, the levels of hydrogen predicted to develop within a few years during peridotite-water interactions greatly exceed the hydrogen yields observed in experiments conducted by Mayhew et al. (2013) at temperatures of 55 and 100°C. These authors suggested that the transfer of electrons from silicates to oxides is the rate-limiting step. If so, then apparently the dissolution of olivine may, though part of reaction sequence, not control the rate of this overall transfer. More experiments like those by Mayhew et al. (2013) will be required to decipher the pathways and rates of hydrogen generating reactions. In the meantime, I suggest that simplified kinetic–thermodynamic modeling may provide useful insights that go beyond what can be achieved by equilibrium thermodynamic computations.

The computational results presented above suggest that the nature of the Fe(III) bearing minerals (e.g., hematite vs. goethite vs. magnetite vs. cronstedtite) is of secondary importance in terms of hydrogen yields at low temperatures when dissolution of olivine is the rate-limiting step (**Figure 4**). This result is surprising, because it is commonly assumed that serpentinization of olivine and production of magnetite are required to have high hydrogen yields. Of course, the equilibrium hydrogen concentrations are greatest, when the fluid equilibrates with magnetite, brucite, and serpentine. But other reaction pathways that lead to the production of more oxidized phases like hematite and goethite are apparently able to produce similar quantities of hydrogen as magnetite, as long as the pace of the reaction is determined by the rate of olivine dissolution. On the other hand, should the rates of Fe(III)-mineral precipitation be slower than the rate of olivine dissolution, then the magnitude of the difference in the rates should play a dominant role in controlling hydrogen production. Figuring out those relative rate differences in the critical reaction steps is an import challenge for future experimental work.

The computations also show that when olivine in a basalt dissolves at a rate similar to basalt glass, it does not develop the same reducing power as it does during serpentinization, because ferroan smectite – and not serpentine + ferric oxide – will form. Only if olivine reacts at rates that are an order of magnitude greater than those of the other phases will olivine-bearing basalts yield more hydrogen than basalt free of olivine. These results lend tentative support to the proposal by Lever et al. (2013), who suggested that alteration of olivine may have played an important role in producing hydrogen for sulfate reducing bacteria in the Juan de Fuca ridge flank system. However, better constraints on the relative rates of alteration of basalt glass and olivine are required to further test this idea.

The computations presented indicate that fresh basalt reacting with seawater may yield micromolal quantities of hydrogen at somewhat elevated temperatures and when water-to-rock ratios are small (e.g., 10). The quantities of hydrogen analyzed in the 60°C Juan de Fuca system (1 μM; Boettger et al., 2013) can easily by produced in basement that is largely isolated from the open ocean, even at temperatures below 60°C (**Figure 5**). Hydrogen production in basaltic ridge flank settings can yield levels high enough to support microbial life (i.e., quantities of several nanomoles per kg water; e.g., Hoehler et al., 2001), if time-integrated water-to-rock ratios are <1000 (by mass). We can assess what this flux number implies in terms of alteration temperature, because we have a tight constraint on the amount of heat (about 7TW) that is lost by circulation of seawater in ridge flanks (Stein and Stein, 1994). Most of that circulation takes place within the uppermost 300 m of basement where permeability is high enough to facilitate fluid flow in the absence of large pressure gradients (Fisher and Becker, 2000). Given that 3 km^2^ of seafloor are generated anually, roughly 2^∗^10^12^ kg yr^−1^ of basaltic upper basement is newly subjected to ridge flank circulation. If this basement is exposed to a 1000-fold greater mass flux of seawater in its lifetime, we arrive at 2 ^∗^ 10^15^ kg of circulating seawater per year. For this mass flux (F), the circulating water must be heated by 21°C (ΔT) to transport 7 TW of heat (Q), given a heat capacity of water (Cp) of 4184 J kg^−1^ °C^−1^ (F = Q/(Cp ^∗^ ΔT); Elderfield and Schultz, 1996). Smaller fluxes of water would come with higher hydrogen concentrations in basaltic ridge flank systems, e.g., at a w/r of 100 (**Figure 8**), but then ΔT would have to be an unreasonable 210°C to account for the 7TW heat loss at a global scale. These results lead me to suggest that hydrogen generation in basaltic ridge flank crust on a global scale is negligible, although it may happen regionally in virtually closed circulation systems (e.g., at the eastern flank of the Juan de Fuca Ridge). Oxidatively altered basalt has an even lower potential of yielding hydrogen by hydrolysis reactions, but radiolytic hydrogen production may play a role in the maturing ocean crust (Türke et al., 2015; Dzaugis et al., 2016).

How do these new assessments influence the validity of the bioenergetic calculations presented by Bach and Edwards (2003)? The iron oxidation rate estimated by these authors (1.7 ± 1.2^∗^10^12^ moles yr^−1^) was recently confirmed by a newer compilation of iron redox state in upper ocean crust (Rutter, 2015). The bioenergetics parameter, e.g., a Δ_r_G of 66 kJ mol^−1^ for iron oxidation and 292 kJ gC^−1^ cellular carbon are also still valid. Bach and Edwards (2003) assumed that only half of the ferrous iron was oxidized to ferric iron by oxygen, the other half, they suspected, is oxidized by water and releases hydrogen. These authors calculated that an energy flux of 0.85^∗^10^12^ moles yr^−1^^∗^ 66 kJ mol^−1^ = 6 ^∗^ 10^13^ kJ yr^−1^ can fix 2 ^∗^ 10^11^ gC biomass yr^−1^. The remaining Fe, they assumed, gives rise to the generation of 0.45 ^∗^ 10^12^ moles of hydrogen yr^−1^, which could support twice the amount of biomass by iron reduction.

**FIGURE 9:**
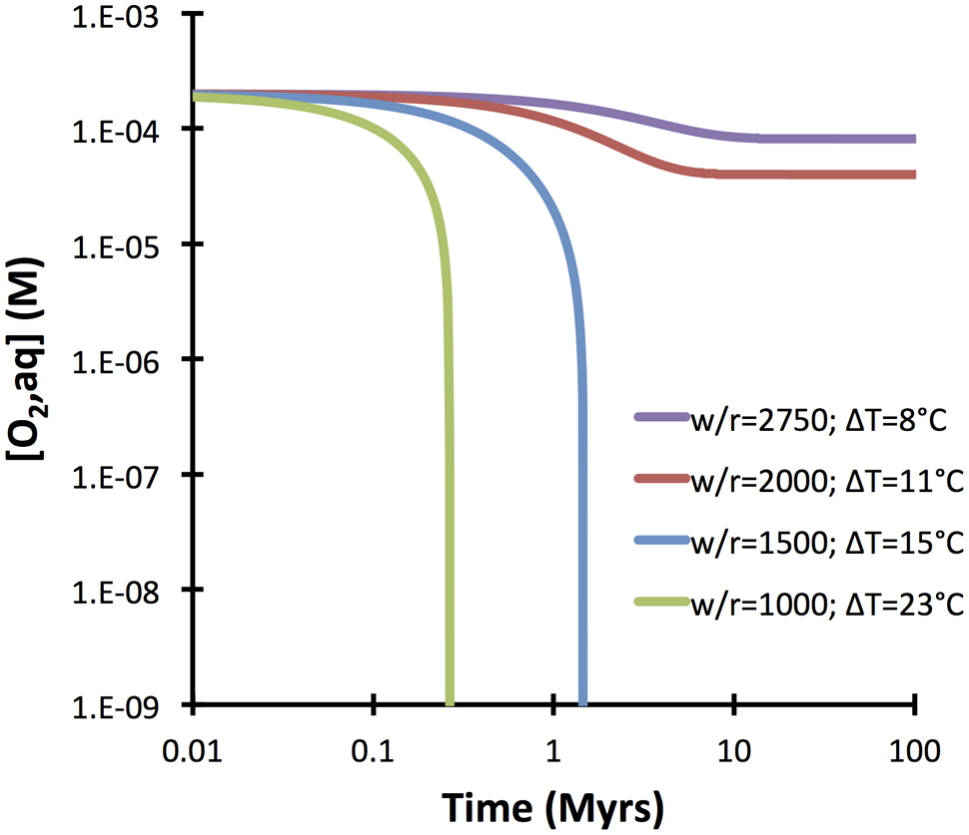
**Predicted evolution of dissolved oxygen in the course of water–basalt interactions at different water-to-rock ratios**. These ratios correspond to temperature anomalies (ΔT) required for circulating seawater in order to account for a global heat flow of 7 TW in ridge flanks. The computations were conducted with GWB for temperatures as indicated in the inset. Note that warm flanks with decreased ventilation by seawater are predicted to turn anoxic after a few million years. Ridge flanks with higher flux rates of seawater (w/r > 2000), in contrast, may stay oxygenated throughout their evolution.

I suggest considering those 50% of oxidation of ferrous iron by water as absolute maximum value. The computations presented above indicate that hydrogen production from hydrolysis may actually be quite minimal in open ridge flank systems, where time-integrated fluid fluxes are large. The North Pond system in the western flank of the mid-Atlantic Ridge is such an open system, where hydrogen in basement fluids is <1 nM and oxygen is still present, although it is respired along the flow path of seawater (Orcutt et al., 2013). In those kind of systems, iron oxidation by oxygen plays a much larger role, and at time-integrated water-to-rock ratios of >2000 (corresponding to a ΔT of 11°C), oxygen is not even the restricting compound (**Figure 9**). In other words, there is more oxygen fluxed those open ridge flanks than is required to account for the amount of Fe oxidation observed. If we assume that all iron in basaltic basement was oxidized by oxygen, the amount of biomass potentially fixed by iron oxidation in basaltic ridge flank aquifers is up to two times higher (i.e., 4 ^∗^ 10^11^ gC biomass yr^−1^) than suggested before. In computing the standing stock of cells (using a maintenance energy of 200 J gC^−1^ yr^−1^ and a cell weight of 26 fg; Whitman et al., 1997) that could be supported by the maximum energy flux related to aerobic iron oxidation (12 ^∗^ 10^13^ kJ yr^−1^), we arrive at 6 ^∗^ 10^14^ gC or 2.4 ^∗^ 10^28^ cells. This is as much as 10% of the sedimentary biomass (Kallmeyer et al., 2012), indicating that iron oxidation in the basaltic aquifer remains one of key potential drivers supporting microbial communities in the basaltic basement.

Hydrogen plays a role where ultramafic and other olivine-rich rocks interact with seawater. This is case in oceanic detachment fault setting, transform faults, bend faults, and in sedimented ridge flanks such as the eastern of the Juan de Fuca Ridge are the Costa Rica Rift Zone. Globally, a hydrogen-fuelled biophere in the oceanic basement may be as large (or larger) as the cold basaltic ridge flank system that runs primarily on Fe-oxidation. In the aging basaltic ridge flanks, Fe oxidation will be slowed as alteration rinds armor the fresh glass and slow down reactions. Strong enrichments of U and K in the alteration rinds, however, give rise to hydrogen production from radiolysis, which may become increasingly important relative to Fe-oxidation in old seafloor (Türke et al., 2015). For a better assessment of the size of a potential hydrogenotrophic subseafloor biosphere, a more detailed understanding of the critical reaction rates and pathways is needed.

## Author Contributions

The author confirms being the sole contributor of this work and approved it for publication.

## Conflict of Interest Statement

The author declares that the research was conducted in the absence of any commercial or financial relationships that could be construed as a potential conflict of interest.
